# Utility of electrocardiogram to predict the occurrence of the no-reflow phenomenon in patients undergoing primary percutaneous coronary intervention (PPCI): a systematic review and meta-analysis

**DOI:** 10.3389/fcvm.2023.1295964

**Published:** 2024-01-08

**Authors:** Elmira Jafari Afshar, Niloofar Gholami, Parham Samimisedeh, MohammadHossein MozafaryBazargany, Amirhossein Tayebi, Amirhossein Memari, Shahrooz Yazdani, Hadith Rastad

**Affiliations:** ^1^ Cardiovascular Research Center, Alborz University of Medical Sciences, Alborz, Iran; ^2^ Rajaie Cardiovascular Medical and Research Center, Iran University of Medical Sciences, Tehran, Iran

**Keywords:** coronary no-reflow, ECG, electrocardiogram, no-reflow phenomenon, percutaneous coronary intervention, PPCI

## Abstract

**Background:**

The no-reflow phenomenon affects about one out of five patients undergoing Primary Percutaneous Coronary Intervention (PPCI). As the prolonged no-reflow phenomenon is linked with unfavorable outcomes, making early recognition is crucial for effective management and improved clinical outcomes in these patients. Our review study aimed to determine whether electrocardiogram (ECG) findings before PCI could serve as predictors for the occurrence of the no-reflow phenomenon.

**Methods and materials:**

We systematically searched MEDLINE, Scopus, and Embase to identify relevant studies. The random-effect model using inverse variance and Mantel-Haenszel methods were used to pool the standardized mean differences (SMD) and odds ratios (OR), respectively.

**Result:**

Sixteen eligible articles (1,473 cases and 4,264 controls) were included in this study. Based on our meta-analysis of baseline ECG findings, the no-reflow group compared to the control group significantly had a higher frequency of fragmented QRS complexes (fQRS) (OR (95% CI): 1.35 (0.32–2.38), *P*-value = 0.01), and Q-waves (OR (95% CI): 1.97 (1.01–2.94), *P*-value <0.001). Also, a longer QRS duration (QRSD) (SMD (95% CI): 0.72 (0.21, 1.23), *p*-value <0.001) and R wave peak time (RWPT) (SMD (95% CI): 1.36 (0.8, 1.93), *P* < 0.001) were seen in the no-reflow group. The two groups had no significant difference regarding *P* wave peak time (PWPT), and *P* wave maximum duration (Pmax) on baseline ECG.

**Conclusion:**

Our findings suggest that prolonged QRSD, delayed RWPT, higher fQRS prevalence, and the presence of a Q wave on baseline ECG may predict the occurrence of the no-reflow phenomenon in patients undergoing PPCI.

## Introduction

Although Primary Percutaneous Coronary Intervention (PPCI) is the method of choice to revascularize the infarct-related artery (IRA) in patients with ST-segment elevation myocardial infarction (STEMI), its efficacy to reestablish myocardial reperfusion may be limited in patients who develop the no-reflow phenomenon (1, 2).

The no-reflow phenomenon is characterized by insufficient myocardial reperfusion despite patent coronary arteries, and it is reported in up to 20% of patients undergoing PPCI (3). This phenomenon can worsen the prognosis in affected patients by increasing the risk of severe left ventricular dysfunction, cardiogenic shock, fatal arrhythmias, and mortality (1).

Its exact mechanism is still unknown, but distal artery embolism, ischemic and reperfusion injury, endothelial dysfunction, and inflammation are suggested to play a role in no-reflow phenomenon pathophysiology (3–5).

Despite recent advances in prevention, diagnosis, and treatment in patients with coronary no-reflow, its management remains challenging for interventional cardiologists (6, 7).

Identifying predictive factors of the no-reflow phenomenon following PPCI could culminate in establishing timely preventive and management techniques and reduce the severity and adverse effects. In this regard, recent studies put a value on electrocardiography (ECG) as an accessible and non-invasive tool employed to predict the no-reflow phenomenon (6, 8, 9); our study aims to summarize and make a comprehensive review of the available evidence on the predictive role of ECG for no-reflow phenomenon following PPCI.

## Methods and materials

This systematic review and meta-analysis followed the Preferred Reporting Items of Systematic Reviews and Meta-analyses (PRISMA) guidelines (10). Since our study was a systematic review of previously published studies, no institutional ethics committee approval was required. All studies that investigated ECG features in patients with the no-reflow phenomenon undergoing PPCI were included.

### Search strategy

To search for relevant studies, three online databases, including PubMed, Scopus, and Embase were systematically searched up to April 10, 2023, using the following keywords in two domains:

1) “No-reflow phenomenon,” “Coronary no-reflow,” “Microvascular obstruction.”
2) “Electrocardiography,” “ECG”

The key terms within each domain were connected using the Boolean operator “OR,” and the two domains were combined using the operator “AND”, adapted for each database.

We also screened the reference lists of related articles, and 100 pages of Google Scholar survey to ensure we did not miss any additional citations. The detailed search strategy is presented in Supplementary Table 1.

### Study selection

Two researchers independently screened the imported articles’ titles, abstracts, and full texts to identify eligible articles. A third senior researcher (H.R.) resolved any disagreements.

### Inclusion criteria

- Observational studies written in English that evaluated ECG characteristics in patients with the no-reflow phenomenon who underwent PPCI for ST-elevation myocardial infarction (STEMI).
- Studies that compared ECG patterns between patients with the no-reflow phenomenon and their controls. The control group should consist of STEMI patients who underwent PPCI without experiencing coronary no-reflow.

### Exclusion criteria

- Animal studies or in-vitro experiments.
- Review articles, commentaries, and opinions.

### Data extraction

Two researchers reviewed the full text of the included articles and extracted data using a standardized data extraction form in Microsoft Excel (Version 2016, Microsoft Corp., Redmond, WA, USA). The extracted data included the first author's name, study design, year, country of origin, sample size, age, gender, cardiovascular disease risk factors and comorbidities, left ventricular ejection fraction (LVEF), culprit vessels, ECG's reported features including *P*-wave maximum duration (*P* max), *P*-wave peak time (PWPT), R-wave peak time (RWPT), QRS duration (QRSD), fragmented QRS (fQRS), Q-wave presence, and the number of leads with Q-waves.

### Risk of bias assessment

The quality of the included studies was evaluated by two trained researchers using the Newcastle-Ottawa Scale (NOS) critical appraisal tool. This tool comprises eight items in three domains, with a total score ranging from 0 to 9, and is recognized for its reliability and validity in assessing the quality of observational studies. Any discrepancies were resolved through discussion between the two researchers.

### Statistically analysis

Our primary objective was to compare ECG parameters between the no-reflow and control groups using a meta-analysis standard method. In this regard, we utilized either the random-effect or fixed-effect models based on the heterogeneity size of the Standardized Mean Differences (SMD) and Odds Ratios (OR). The magnitude and significance of the heterogeneity were determined by *I*-square statistics and Q-test, respectively. If the *I*-square was greater than 50% or the *P*-value was less than 0.1, we used the random-effect model. We combined SMDs and ORs using inverse variance and Mantel-Haenszel methods, respectively.

We computed the SMD for some ECG features, including the mean differences of *P* max, PWPT, the number of leads with Q waves, QRSD, RWPT, as well as the crude OR for fQRS and Q wave between the no-reflow and control groups. Meta-analyses were performed using the R Meta package in R Studio software (version 4.3.1.).

## Result

### Study selection process

Our comprehensive search of electronic databases yielded 1,698 documents. After excluding duplicates (*N* = 420) and irrelevant items (*N* = 1,221), 16 articles (comprising 1,473 cases and 4,264 controls) met our eligibility criteria and were included in this review (Supplementary Figure 1).

### Characteristics of included studies

Included studies were published since 2001, mainly in Turkey (*N* = 9), followed by India (*N* = 2), Japan (*N* = 2), Iran (*N* = 1), Egypt (*N* = 1), and Indonesia (*N* = 1).

After conducting a quality assessment using the Newcastle-Ottawa Scale (NOS) critical appraisal tool, we found that all studies included in our analysis scored between six to nine points, indicating a high level of quality across the studies (Supplementary Table 2).

Based on our pooled analysis, there was a comparable proportion of male individuals in both groups (78% vs. 81.9%). Additionally, the pooled mean (standard deviation) age was roughly similar between the two groups [60.5 (11.8) vs. 57 (11.3)]. The prevalence of smoking (56.4% vs. 45.8%), hypertension (42.5% vs. 39.7%), diabetes mellitus (31% vs. 22.6%), and dyslipidemia (41.5% vs. 33.6%) were comparable between the no-reflow and the reflow groups. The left anterior descending artery (LAD) was the culprit artery in over 60% of cases in both groups (62.1% vs. 61.2%). Interestingly, in all eight studies reporting left ventricular ejection fraction (LVEF), the no-reflow group exhibited a significantly lower LVEF compared to the control group (Table 1).

**Table 1:** Characteristics and main findings of included studies.

ID	Author, year	Country	Groups	N	Male, % (*N*)	Age, year, mean (SD)	Cardiovascular risk factors, % (*N*)				Culprit's vessels % (*N*)			Anterior STEMI**%**	LVEF%	^a^ECG findings
							DM	HTN	DLP	Smoking	LAD	RCA	LCX			
1	Cagdas et al. (8)	Turkey	Case	99	74.7* (72)	65 (11)	45.4* (45)	54.5* (54)	33.3 (33)	66.7* (66)	NR	NR	NR	NR	↓*	↑ pre RWPT* /↑ post RWPT* ↑ pre QRSD*/↑ post QRSD* ↑ Q wave presence* ↓ STR* ↑ post ∑STE*,∼pre-STE
			Control	134	83.6* (112)	61 (12)	22.4* (30)	37.3* (50)	23.9 (32)	48.5* (65)	NR	NR	NR	NR		
2	Maden et al. (11)	Turkey	Case	26	80.8 (21)	62.7* (14.2)	35 (9)	42 (11)	54 (14)	NR	17 (65)	7 (27)	2 (8)	65	NR	↑ pre QRSD*/↑ post QRSD* ↑ 60 min QRSD/↓ 60 min QRS narrowing ∼ pre-STE
			Control	136	88.2 (120)	54.7* (13.5)	15 (21)	32 (43)	NR	62 (85)	57 (77)	34 (47)	12 (9)	57		
3	Karahan et al. (12)	Turkey	Case	105	87.6 (92)	57 (11)	8.6 (9)	22.8 (24)	NR	47.6 (50)	61 (58.1)	17.1 (18)	24.8 (26)	59	↓*	∼ pre-QRSD/↑ post-QRSD* ↑ 60 min QRSD*
			Control	108	77.8 (84)	58.3 (12)	16.7 (18)	25 (27)	NR	38.9 (42)	42.6 (46)	13 (12)	45.4 (49)	43.5		
4	Bendary et al. (13)	Egypt	Case	39	74.4 (29)	56 (9)	51.4* (20)	64.1* (25)	71.8 (28)	66.7* (26)	69.2 (27)	23.1 (9)	7.7 (3)	69	↓*	↑ Q wave presence* ↑ max STE*/↓ single lead STR* ↑ pre QRSD*/↑ post QRSD* ↑ pre RWPT*/↑ post RWPT*
			Control	61	70.5 (43)	52 (12)	29.9* (18)	39.3* (24)	75.4 (46)	34.4* (21)	54.1 (33)	32.8 (20)	13.1 (8)	62.3		
5	Yusuf et al. (14)	India	Case	70	70* (49)	58.3* (11.2)	35.7* (25)	34.3 (24)	38.6 (27)	33 (47.1)	58.6 (41)	34.3 (24)	7.1 (5)	53	↓*	↑ pre QRSD* ↑ post QRSD* ∼ pre-STE /↑ post-STE* ↑ pre RWPT*/↑ 60 min RWPT* ↓ STR >50%*/↓ STR > 70%* ↑ Q wave presence
			Control	130	86.2* *(112)	51* (8.8)	21.5* (28)	24.6 (32)	33.1 (43)	50 (65)	50.8 (66)	40 (52)	9.2 (12)	56		
6	Yusuf et al. (15)	India	Case	80	95 (76)	49 (13.1)	17.5 (14)	20 (16)	NR	NR	71.2 (57)	18.8 (15)	10 (8)	71.2	↓*	∼ pre-QRSD ↑ post-QRSD* ↑ 60 min QRSD* ↓ STR*
			Control	120	89.2 (107)	47.7 (9.3)	18.3 (22)	22.5 (27)	NR	NR	58.3 (70)	26.7 (32)	15 (18)	58.3		
7	Suzuki et al. (16)	Japan	Case	6	100 (6)	65 (13)	NR	NR	NR	NR	NR	NR	NR	NR	NR	↑ pre QRSD* ∼ pre-aVR STE/∼ pre-STE/∼ pre-ST deviation ∼ VF or VT
			Control	10	70 (7)	70 (9)	NR	NR	NR	NR	NR	NR	NR	NR		
8	Ketaren et al. (17)	Indonesia	Case	10	90 (9)	63.9* (12.2)	30 (3)	70 (7)	80 (8)	40 (4)	40 (4)	40 (4)	20 (2)	10	NR	↑ pre QRSD
			Control	31	90 (27)	53.7* (10.3)	22.6 (7)	48.4 (15)	80.6 (24)	64.5 (19)	48.4 (15)	45.2 (14)	6.4 (2)	6.5		
9	Hayıroğlu et al. (18)	Turkey	Case	32	81.2* (26)	65 (10)	34.4 (11)	62.5* (20)	12.5 (4)	50 (16)	100 (32)	NR	NR	100	↓*	∼ pre ∑ precordial STE ∼ pre-QRSD/∼ fQRS presence ↑ Q wave presence* ↑ ∑ precordial Q duration (QD)*/↓ ∑ precordial R duration (RD)*/↑ QD vs. RD*
			Control	371	82.7* (307)	56 (12)	22.4 (83)	35.6* (132)	8.1 (30)	63.6 (236)	100 (371)	NR	NR	100		
10	Cagdas et al. (19)	Turkey	Case	22	72.7 (16)	66 (10)	59.1 (13)	54.5 (12)	NR	63.6 (14)	NR	NR	NR	100	↓*	∼ pre-STE∼pre-max STE ↑ post ∑STE*/↑ post max STE*/↓ STR* ↑ Q wave presence* ↑ pre PWPT_lead (II)_ *_/_ ↑ pre PWPT_lead(V1)_ ↑* post PWPT_lead (II)_*/↑ post PWPT_lead(V1)_*
			Control	34	70.6 (24)	64 (13)	41.2 (14)	47.1 (16)	NR	52.9 18)	NR	NR	NR	100		
11	Karakurt et al. (20)	Turkey	Control	110	70.9 (78)	67.2* (11.8)	44.6* (49)	52.7 (58)	39.1 (43)	58.2* (64)	30.9 (50)	54.9 (89)	11.7 (19)	30.9	↓*	∼ pre-Pmax /↑ post P_max_* ∼ pre-*P*_min_ ↑ post P_min_*∼pre-Pd /↑ post Pd* ∼ pre-max STE ↑ post max STE
			Case	162	126 (77.8)	63.6* (2.5)	20.7* (33)	45.1 (73)	30.2 (49)	46.3* (75)	49.1 (54)	31.8 (35)	10 (11)	49.1		
12	Alidoosti et al. (21)	Iran	Control	166	81.9 (136)	59.9* (12.3)	28.9 (48)	51.8 (86)	57.9 (95)	34.4 (55)	65.1 (108)	22.9 (38)	12 (20)	48.2	NR	↑ Number of the Q wave *
			Case	364	76.9 (280)	57.3* (12.3)	28.6 (104)	60.4 (220)	60.5 (219)	36.6 (132)	50.3 (183)	33.5 (122)	16.2 (59)	37.9		
13	Iwakura et al. (22)	Japan	Case	79	74.7 (59)	60 (10)	20.2 (16)	23.4 (18)	16.5 (13)	25.3 (20)	NR	NR	NR	100	NR	↑ Number of Q waves* ∼ transient ST re-elevation
			Control	120	82.5 (99)	58 (10)	23.3 (28)	24.1 (29)	15 (18)	30 (36)	NR	NR	NR	100		
14	Acar et al. (23)	Turkey	Case	56	75* (42)	64.4* (13.6)	26.8 (15)	44.6 (25)	NR	46.4 (26)	NR	NR	NR	100	NR	↑ fQRS*
			Control	203	87.7* (178)	60.2* (12.6)	19.8 (40)	46.8 (95)	NR	55.7 (113)	NR	NR	NR	100		
15	Ozkan et al. (24)	Turkey	Case	222	NR	NR	NR	NR	NR	NR	NR	NR	NR	NR	NR	↑ fQRS
			Control	179	NR	NR	NR	NR	NR	NR	NR	NR	NR	NR		
16	Kaya et al. (25)	Turkey	Case	351	NR	NR	NR	NR	NR	NR	NR	NR	NR	NR	NR	↑ fQRS
			Control	2,101	NR	NR	NR	NR	NR	NR	NR	NR	NR	NR		
	Pooled	N	Case	1,473	78.2 (704/900)	60.5 (11.8)	31 (277/894)	42.5 (380/894)	41.5 (251/605)	56.4 (459/814)	62.1 (429/691)	32.2 (197/612)	13.6 (83/612)	NR	–	**–**
			Control	4,264	81.9 (1,626/1,984)	57.0 (11.3)	22.6 (447/1,974)	39.7 (783/1,974)	33.6 (461/1,373)	45.8 (850/1,854)	61.2 (898/1,467)	21.4 (288/1,347)	11.8 (159/1,347)	NR	**–**	**–**

DLP, Dyslipidemia; DM, Diabetes mellitus; fQRS, Fragmented QRS; HTN, Hypertension; LAD, Left anterior descending; LCX, Left circumflex; LVEF, Left Ventricular Ejection Fraction; MI, Myocardial infarction; *N*, Number; NR, Not reported; *P* max, *P*-wave maximum duration; *P* min, *P*-wave minimum duration; Pd, *P*-wave dispersion; RCA, Right coronary artery; RWPT, R-wave peak time; SD, Standard deviation; STEMI, ST-elevation myocardial infarction; TFC, TIMI frame count.

^a^ The arrow direction shows significant differences in ECG findings between no-reflow patients and controls, with upward arrows indicating higher values in no-reflow patients and downward arrows indicating lower values.

* Shows the differences between case and control groups are statistically significant (*P* value <0.05).

### Qualitative synthesis

Consistently across the included studies, the observed differences in ECG features between patients with and without the no-reflow phenomenon consistently aligned in the same direction. Among the eight studies that compared QRSD between the two groups, seven of them reported a significantly higher QRSD in the no-reflow groups compared to the control group (7/8) (11, 12, 14–16, 18, 23). All three studies that evaluated the frequency of fQRS consistently reported a significantly higher prevalence of fQRS in the no-reflow group compared to the control group (23–25). The Q-waves on admission were reported in five studies, and in all of them, patients who developed the no-reflow phenomenon had a higher incidence of Q-waves than the control group (8, 14, 19, 26). Furthermore, the number of leads with Q-waves on ECG was significantly higher in the no-reflow group compared to the control group in both studies which reported this parameter (21, 22). RWPT was longer in the no-reflow group than the control group in all studies that evaluated it (*N* = 3) (8, 13, 14). Two studies evaluated the association between longer PWPT and no-reflow phenomenon, and one study found a significant correlation between prolonged PWPT in the no-reflow group compared to the control group but the other one didn't find any significant differences (19, 20). *P*-wave dispersion was evaluated between the no-reflow and control groups in one study which found an insignificant difference between the two groups in the baseline ECG (20). None of the six studies that reported ∑STE found a significant difference between the two groups (8, 14, 16, 19, 20); however, the no-reflow group exhibited significantly lower ST resolution (STR) compared to the control group (*N* = 3) (13, 14, 19). In the one study that evaluated transient ST segment re-elevation, no significant difference was found between the no-reflow group and the control group (22).

### Meta-analysis

**Figure 1:**
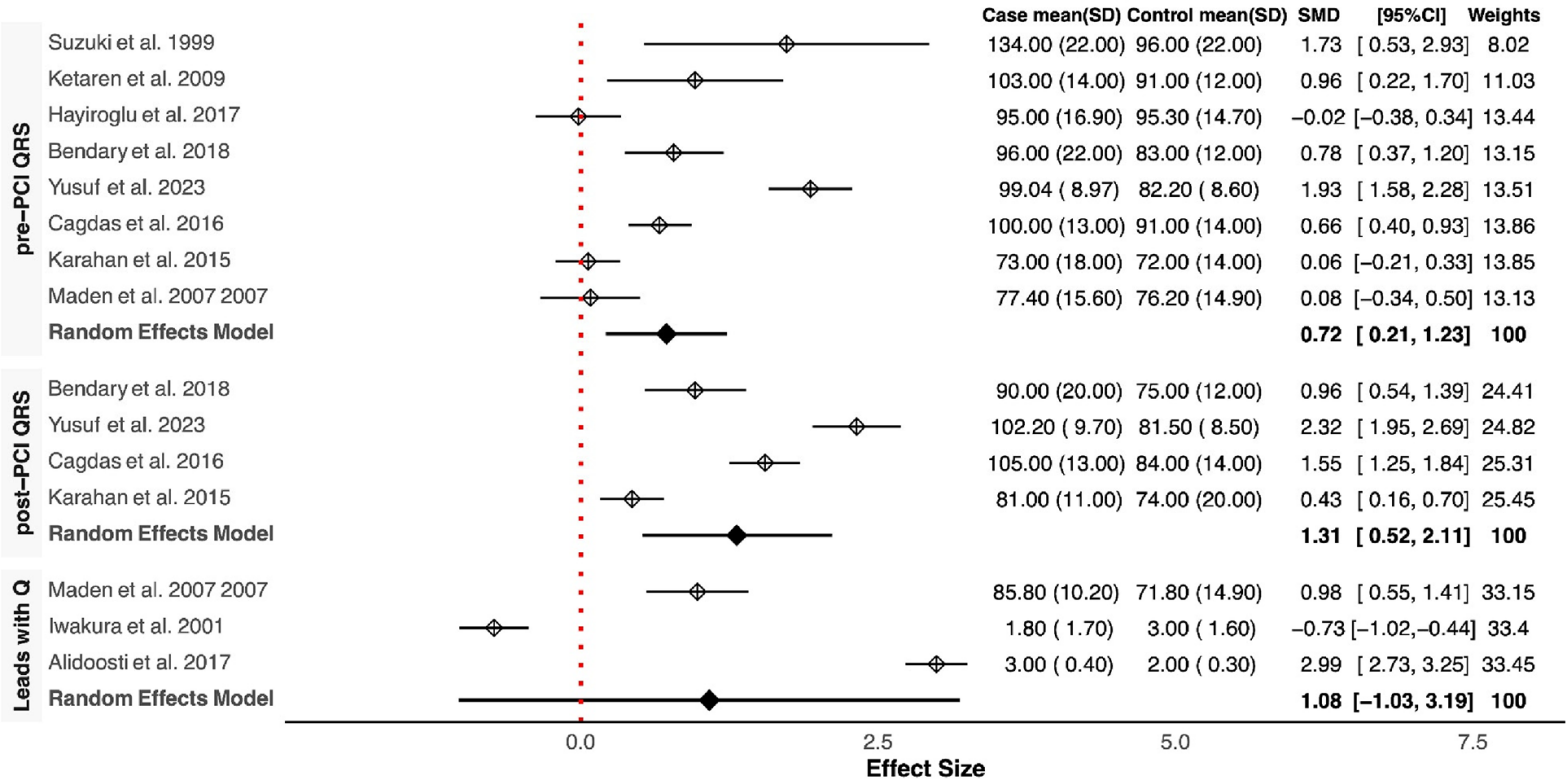
Forest plots showing the standardized mean differences of pre & post QRSD, number of leads with Q wave.

**Figure 2:**
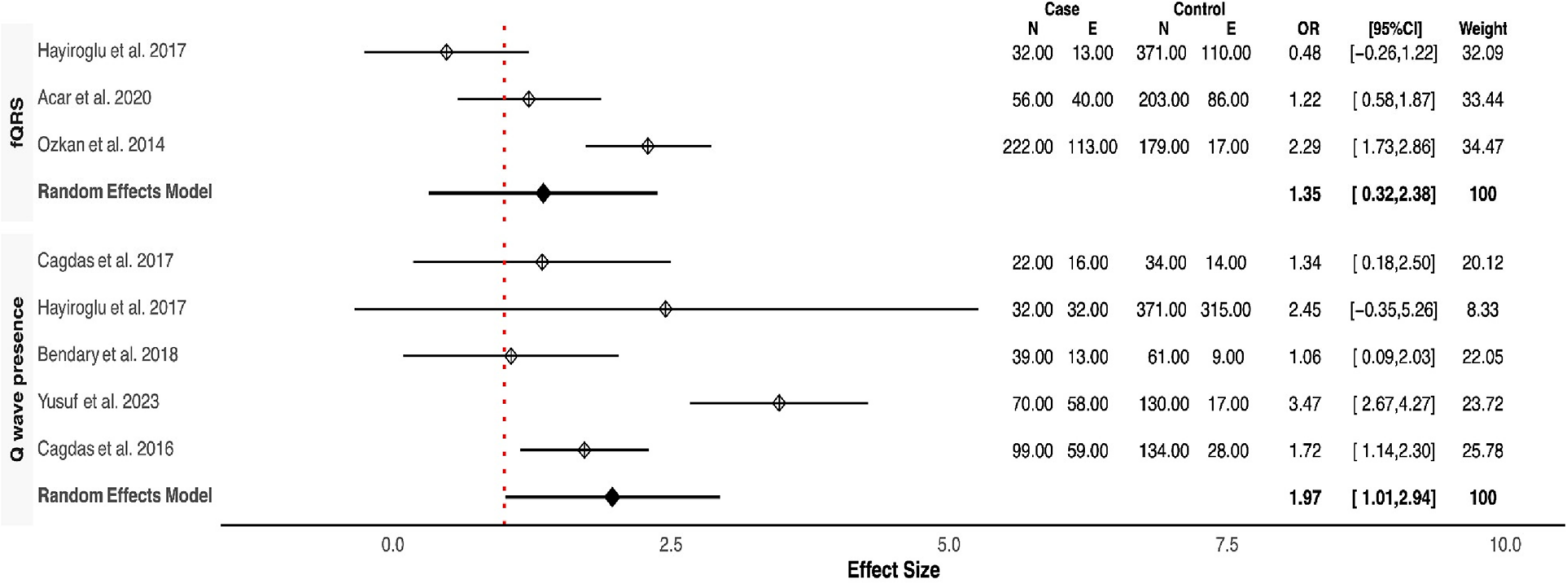
Forest plots showing the odds ratio of fQRS and Q wave presence.

**Figure 3:**
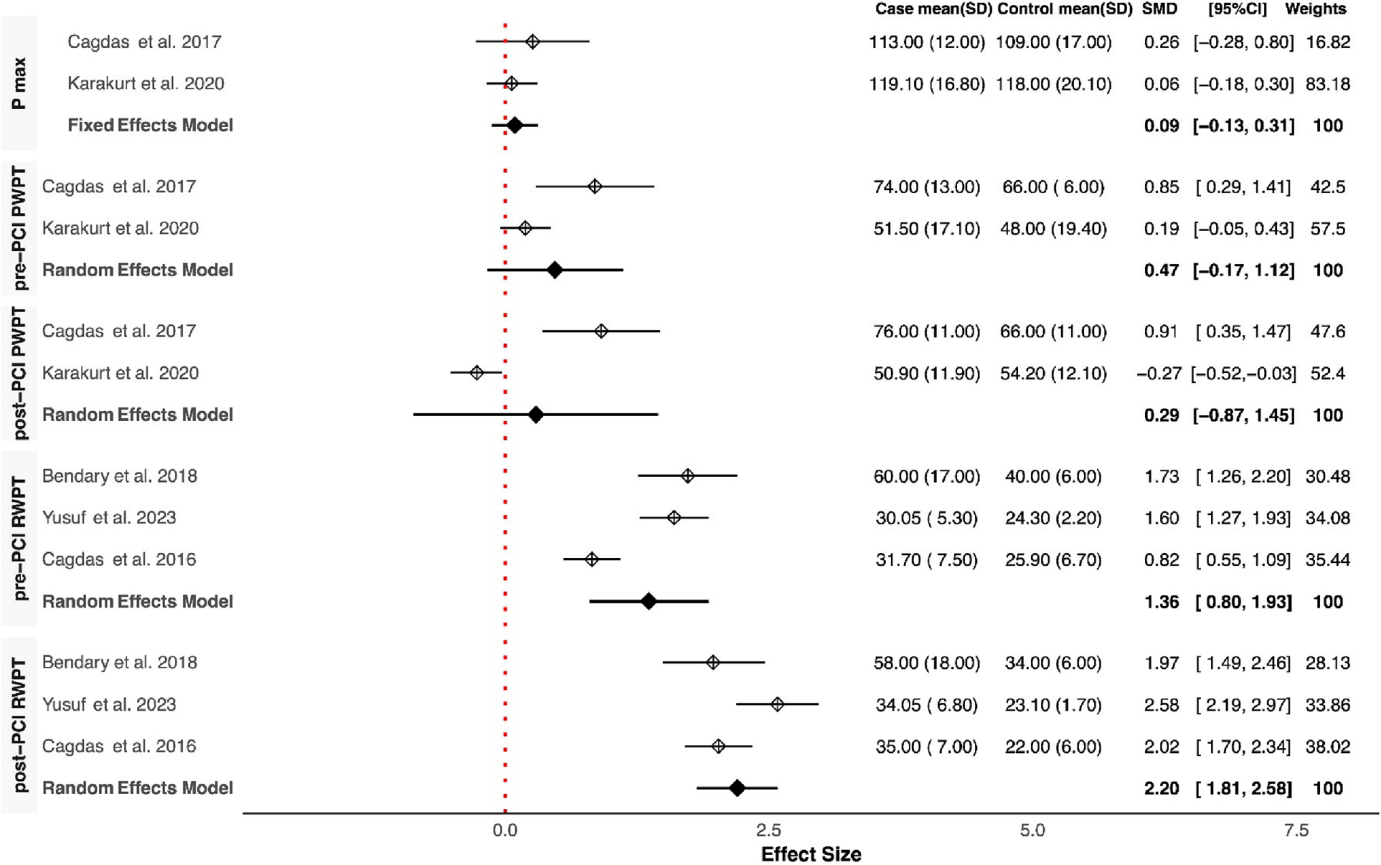
Forest plots showing the standardized mean differences of Pmax, pre & post-PCI PWPT, and pre & post-PCI RWPT.

Our meta-analysis revealed that QRSD was significantly longer in the no-reflow group compared to the control group [SMD (95% CI): 0.72 (0.21–1.23; *P* < 0.001)] (Figure 1). The frequency of fQRS was significantly higher in the no-reflow group [OR (95% CI): 1.35 (0.32–2.38, *P* = 0.01)] (Figure 2). The Q wave was significantly more frequent in the no-reflow in comparison with the re-flow group [OR (95% CI): 1.97 (1.01–2.94, *P* < 0.001)] (Figure 2). Based on our pooled analysis the average number of Q waves in no-reflow patients didn't show significant differences between the reflow and the reflow groups (SMD (95% CI): 1.08 [−1.03,3.19, *P* = 0.54)] (Figure 1). RWPT was significantly longer in the no-reflow group than reflow (SMD [95% CI:1.36 (0.8,1.93, *P* < 0.001)] (Figure 3). In our meta-analysis, there wasn't any significant difference in PWPT between the reflow and the no-reflow phenomenon [SMD (95% CI): 0.47 (−0.17–1.12, *P* = 0.15)]. Pmax had no significant difference in the two groups [SMD (95% CI): 0.09 (−0.13, 0.31, *P* = 0.41)] (Figure 3). We also draw funnel plots to evaluate publication bias for each ECG variable, which are depicted in Supplementary Figures 2–4.

## Discussion

We performed a systematic review and meta-analysis on studies assessing the role of ECG in predicting the occurrence of no-reflow in patients undergoing PPCI. Based on our pooled analysis, no-reflow patients compared to their counterparts had significantly longer QRSD and RWPT on ECG at the baseline. Also, fQRS and the presence of Q waves were more frequently observed in the no-reflow group than in the controls. Regarding Heart rate and Pmax, the difference between the two groups was statistically non-significant.

No-reflow is a common but underestimated complication occurring during or after PCI, particularly PPCI, leading to serious adverse outcomes such as heart failure and cardiac death (2, 27). Multiple factors such as embolization during the percutaneous coronary intervention, the formation of platelet and neutrophil aggregates, vasoconstriction in the microvasculature, and extravascular compression are supposed to be involved in the pathophysiology of the no-reflow phenomenon (28). Identifying the risk factors and predictors for the development of no-reflow can enable early prevention and effective management of patients, ultimately leading to improved clinical outcomes. The presence of certain comorbidities, such as hyperglycemia, hypertension, hypercholesterolemia, renal insufficiency, plaque composition, and high thrombus burden, have been linked to an increased risk of no-reflow phenomena in patients. This association may be attributed to underlying vascular disease, inflammation, and elevated oxidative stress that often accompany these conditions (29). Imaging techniques like contrast-enhanced echocardiography, cardiac MRI, and angiography can be helpful in predicting the onset of the no-reflow phenomenon by providing valuable insights into the severity and extent of myocardial damage and impaired blood flow. Nevertheless, these imaging modalities may not always be easily accessible or readily available (30).

ECG is a simple and non-invasive diagnostic tool widely used in clinical practice. Cohort studies have investigated the value of bassline ECG findings, such as ST-segment elevation, T-wave inversion, and prolonged QT interval, in predicting the no-reflow phenomenon (8).

The exact mechanism that links prolonged QRSD at baseline to the occurrence of the no-reflow phenomenon is not yet fully understood. Prolonged QRS duration is a sign of impaired conduction status of the Purkinje fibers resulting from myocardial damage and scar tissue formation, which are associated with oxidative stress and microvascular dysfunction—factors that contribute to the development of the no-reflow phenomenon (11, 15, 16, 31). Fragmented QRS (fQRS), an abnormal finding on the ECG, is associated with worse outcomes, such as arrhythmias, recurrent myocardial infarction, heart failure, and cardiac death. In patients with STEMI. fQRS may reflect severe myocardial damage and the presence of fibrosis and scar tissue, disrupting normal cardiac conduction (32).

The exact mechanism underlying the association between delayed RWPT on baseline ECG and the occurrence of no-reflow during PPCI has yet to be fully elucidated. However, it has been proposed that delayed RWPT may indicate the presence of late electrical activation of the left ventricle caused by impaired myocardial blood flow (8, 14).

Delayed referral to the hospital following an infarction is a risk factor for the no-reflow phenomenon, which can lead to tissue necrosis and the development of Q waves on ECG. The presence of Q waves indicates conduction abnormalities resulting from transmural extent myocardial infarction or tissue necrosis, which are predisposing factors for the no-reflow phenomenon (21).

Identifying ECG findings associated with the no-reflow phenomenon could have practical implications for clinicians and patient management (2). For instance, some studies have suggested the use of prophylactic vasodilator drugs, such as adenosine, nitrates, and calcium channel blockers, to prevent the occurrence of the no-reflow phenomenon; however, the use of these drugs in all patients as a standard preventive measure is limited due to the potential for adverse events associated with their administration. Early risk stratification based on the no-reflow associated ECG patterns could justify prophylaxis drug administration in high-risk patients (5, 33). Furthermore, device-based techniques, including thrombus aspiration and distal protection, when combined with stenting, have demonstrated a significant reduction in the incidence of the no-reflow phenomenon. However, these techniques are not routinely employed in all PCI procedures (7). By utilizing ECG for early recognition of high-risk patients for the no-reflow phenomenon, clinicians can proactively prepare the cath lab, ensuring the availability of the necessary equipment for these procedures (2, 4).

## Conclusion

The findings of our meta-analysis study suggest that some ECG parameters including prolonged QRS duration, delayed RWPT, and presence of Q-wave may play a role in predicting the occurrence of no-reflow in patients undergoing PCI.

### Limitation and strength

To the best of our knowledge, this is the first systematic review and meta-analysis study that compares ECG features between two groups. However, further studies are needed to confirm our findings and to assess other ECG features.

## Data Availability

The original contributions presented in the study are included in the article/**Supplementary Material**, further inquiries can be directed to the corresponding author/s.
