# Supplementary material for: Utility of electrocardiogram to predict the occurrence of the no-reflow phenomenon in patients undergoing primary percutaneous coronary intervention (PPCI): a systematic review and meta-analysis
Source: Front Cardiovasc Med. 2024 Jan 8;10:1295964. doi: 10.3389/fcvm.2023.1295964 (PMC10813196; doi:10.3389/fcvm.2023.1295964)
Supplement: Supplementary file 1 [file Datasheet1.docx]

**Supplementary Table 1.** Detailed systematic search strategy.

| **Research question** | What are the ECG characteristics of patients undergoing PPCI who developed the no-reflow phenomenon? |
| --- | --- |
| **Research title** | ECG characteristics of patients with the coronary slow flow phenomenon: A systematic review and meta-analysis |
| **PICOT** | **P:** Patients undergoing PPCI who were diagnosed with no-reflow  **E:** ECG characteristics/findings  **C:** Patients undergoing PPCI without no-reflow  **O:** Differences in ECG patterns between the no-reflow and control group  **T:** Observational studies |
| **Keywords** | Coronary No-reflow, No-reflow phenomenon, No-reflow, NRP, impaired myocardial  reperfusion, Electrocardiography, ECG, EKG, P-wave peak time, P-wave dispersion, P-wave  time index, Q/R duration, QRS duration, Fragmented QRS, QT interval, Q-wave  development, T-wave inversion, Loss of R-wave amplitude |
| **Search strategy**  (Searched on 10^th^ April) | **Scopus:** ( ( TITLE-ABS-KEY ( &quot;Coronary no-reflow&quot; ) ) OR ( TITLE-ABS-KEY ( &quot;No-reflow  phenomenon&quot; ) ) OR ( TITLE-ABS-KEY ( &quot;No-reflow&quot; ) ) OR ( TITLE-ABS-KEY (  &quot;Microvascular obstruction&quot; ) ) ) AND ( ( TITLE-ABS-KEY ( &quot;Electrocardiography&quot; ) )  OR ( TITLE-ABS-KEY ( &quot;Electrocardiogram&quot; ) ) OR ( TITLE-ABS-KEY ( &quot;ECG&quot; ) ) OR  ( TITLE-ABS-KEY ( &quot;EKG&quot; ) ) OR ( TITLE-ABS-KEY ( &quot;P-wave&quot; ) ) OR ( TITLE-ABS-  KEY ( &quot;QRS&quot; ) ) OR ( TITLE-ABS-KEY ( &quot;QT&quot; ) ) OR ( TITLE-ABS-KEY ( &quot;T-wave&quot; )  ) OR ( TITLE-ABS-KEY ( &quot;Q-wave&quot; ) ) ) (N=649) |
|  | **PubMed:** (((((&quot;No-Reflow Phenomenon&quot;[Mesh]) OR (&quot;Coronary no-reflow&quot;[Title/Abstract])) OR  (&quot;No-reflow&quot;[Title/Abstract])) OR (&quot;Microvascular obstruction&quot;[Title/Abstract])) OR  (&quot;Impaired myocardial reperfusion&quot;[Title/Abstract])) AND  ((((((((((((&quot;Electrocardiography&quot;[Mesh]) OR (&quot;ECG&quot;[Title/Abstract])) OR  (&quot;EKG&quot;[Title/Abstract])) OR (&quot;P-wave&quot;[Title/Abstract])) OR (&quot;QRS&quot;[Title/Abstract])) OR  (&quot;QT&quot;[Title/Abstract])) OR (&quot;T-wave&quot;[Title/Abstract])) OR (&quot;Fragmented  QRS&quot;[Title/Abstract])) OR (&quot;Q-wave&quot;[Title/Abstract])) OR (&quot;P-wave peak  time&quot;[Title/Abstract])) OR (&quot;R-wave&quot;[Title/Abstract])) OR (&quot;P-wave  dispersion&quot;[Title/Abstract])) (N=410) |
|  | **Embase:** (‘no-reflow OR ‘ coronary no-reflow OR ‘no-reflow phenomenon’ OR ‘microvascular obstruction’ OR ‘impaired myocardial reperfusion’) AND (electrocardiography OR ‘ ECG’ OR ‘EKG’ OR ‘p-wave’ OR ‘qt’ OR ‘t-wave’ OR ‘ fragmented qrs’ OR ‘ q wave’ OR ‘r-wave’)  (N = 639) |

**Supplementary Figure 1:** PRISMA Flowchart of literature search and selection process. [1]


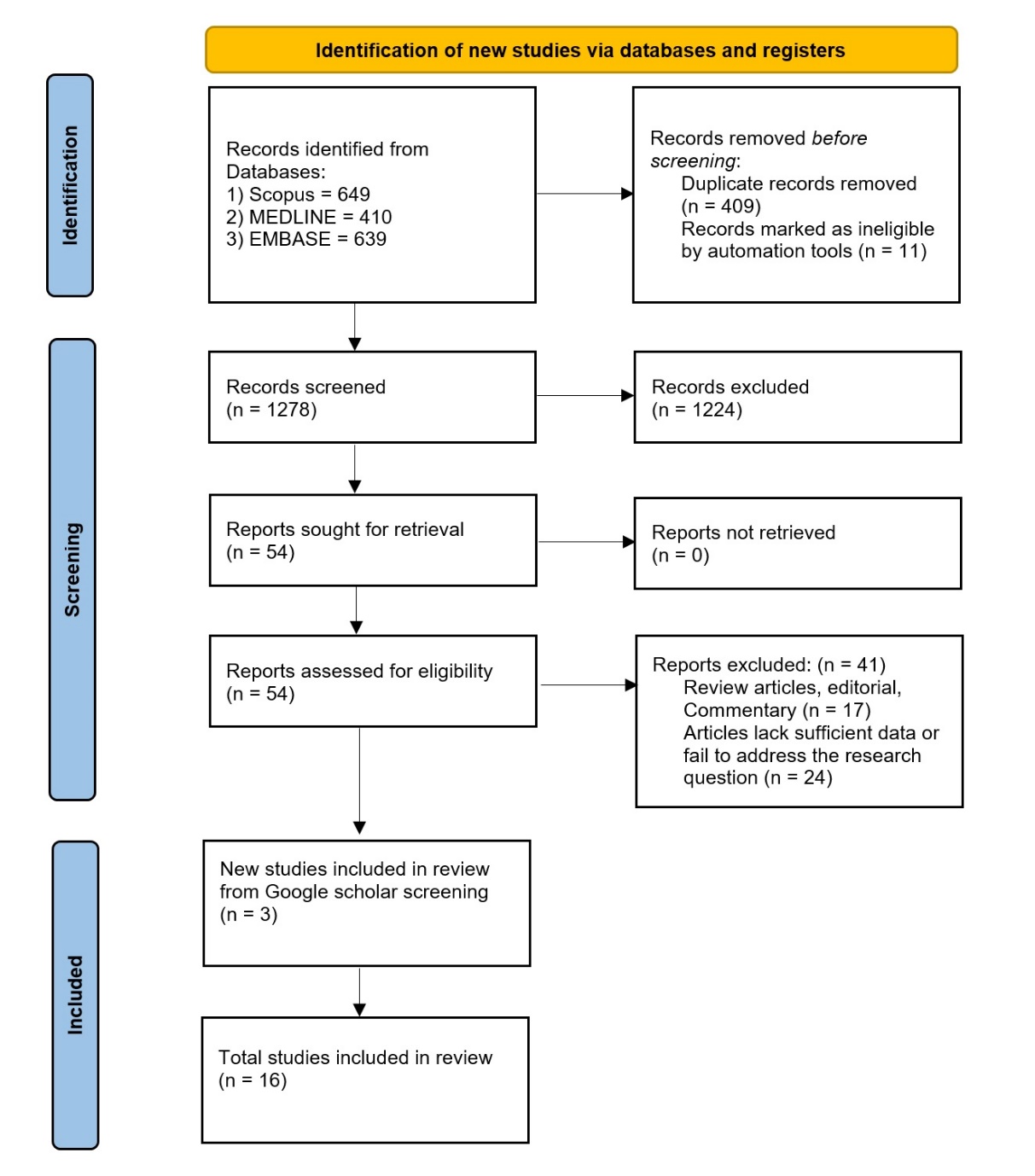


**Supplementary Table 2.** Quality and risk of bias assessment of included studies according to Newcastle-Ottawa Scale (NOS) critical appraisal tool for case-control studies

| N. | Study | Selection | | | | Comparability | Exposure | | | Total  (9/9) | Class |
| --- | --- | --- | --- | --- | --- | --- | --- | --- | --- | --- | --- |
|  |  | 1 | 2 | 3 | 4 | a | I | II | III |  |  |
| 1 | Cagdas et al.  2017 |  | * |  | * | ** | * | * | * | 7/9 | Good |
| 2 | Maden et al.  2008 | * | * |  | * | ** | * | * | * | 8/9 | Good |
| 3 | Karahan et al.  2015 | * | * |  | * | * | * | * | * | 7/9 | Good |
| 4 | Bendary et al.  2018 | * | * |  | * | * | * | * | * | 7/9 | Good |
| 5 | Yusuf et al.  2023 | * | * |  | * | ** | * | * | * | 8/9 | Good |
| 6 | Yusuf et al.  2018 |  | * |  | * | * | * | * | * | 6/9 | Good |
| 7 | Suzuki et al.  2003 |  | * |  | * | ** | * | * | * | 7/9 | Good |
| 8 | Ketaren et al.  2009 | * |  | * | * | * |  | * | * | 6/9 | Good |
| 9 | Hayıroğlu et al.  2017 |  | * |  | * | * | * | * | * | 6/9 | Good |
| 10 | Cagdas et al.  2017 | * | * |  | * | ** | * | * | * | 8/9 | Good |
| 11 | Karakurt et al.  2020 |  | * |  | * | * | * | * | * | 6/9 | Good |
| 12 | Alidoosti et al.  2017 | * | * |  | * | ** | * | * | * | 8/9 | Good |
| 13 | Iwakura et al.  2001 | * | * |  | * | ** | * | * | * | 8/9 | Good |
| 14 | Acar et al.  2021 | * | * |  | * | ** | * | * | * | 8/9 | Good |
| 15 | Ozkan et al.  2014 | * | * |  | * | ** | * | * | * | 8/9 | Good |
| 16 | Kaya et al.  2019 | * | * |  | * | ** |  | * | * | 7/9 | Good |

1: Is the case definition adequate? (*)

2: Representativeness of the cases (*)

3: Selection of Controls (*)

4: Definition of Controls (*)

a: Comparability of cases and controls on the basis of the design or analysis (**)

I: Ascertainment of exposure (*)

II: Same method of ascertainment for cases and controls (*)

III: Non-Response rate (*)

**Supplementary Figure 2.** Funnel plots for publication biases Leads with QRS, Post-PCI- R wave peak time, and Pre-PCI- R wave peak time.


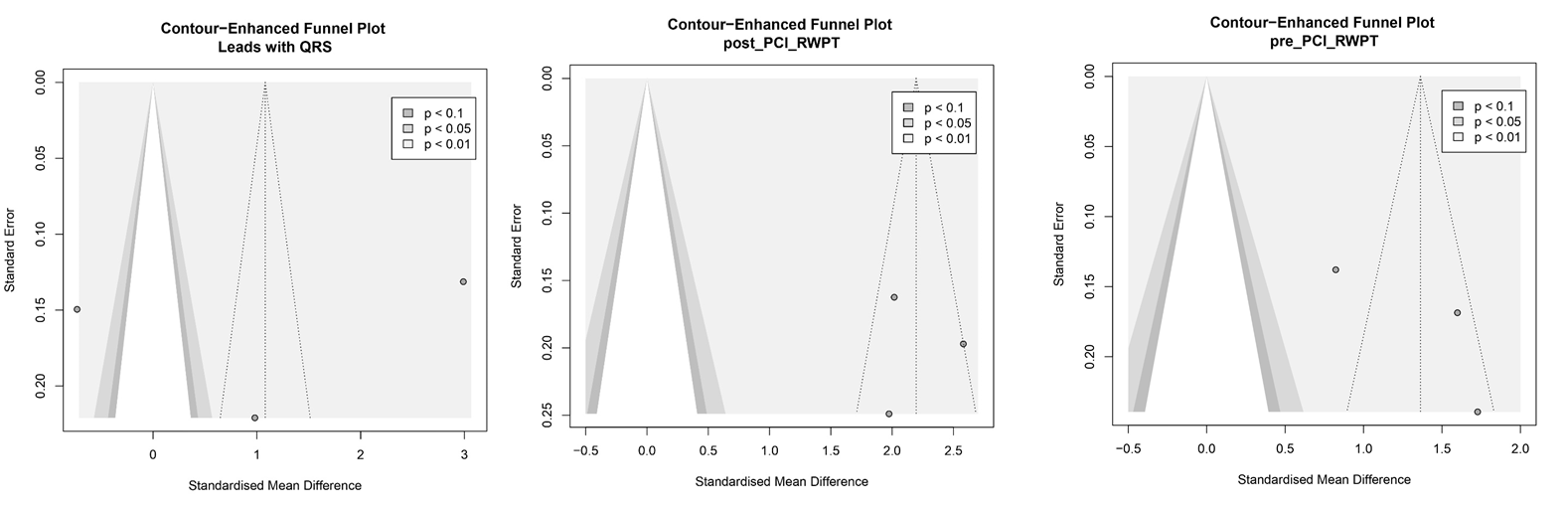


**Supplementary Figure 3.** Funnel plots for publication biases P max, Post-PCI- P wave peak time, and Pre-PCI- P wave peak time.

**
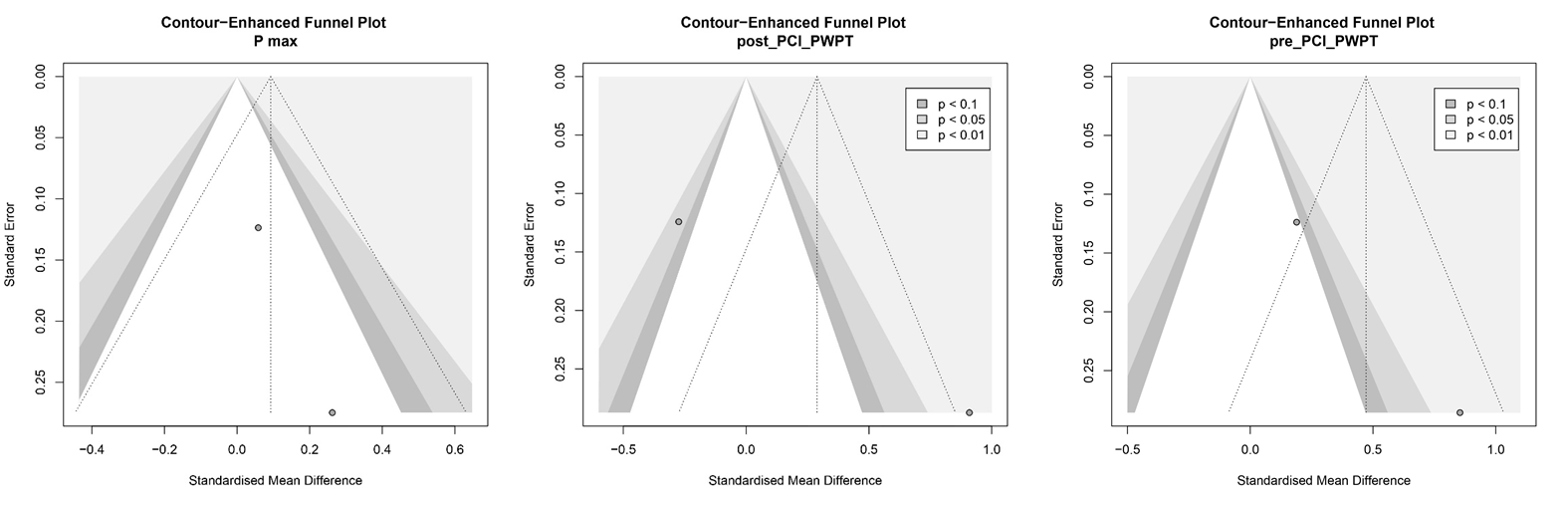
**

**Supplementary Figure 4.** Funnel plot for publication biases Pre and Post-PCI QRS duration and presence of Q-wave in ECG.

**
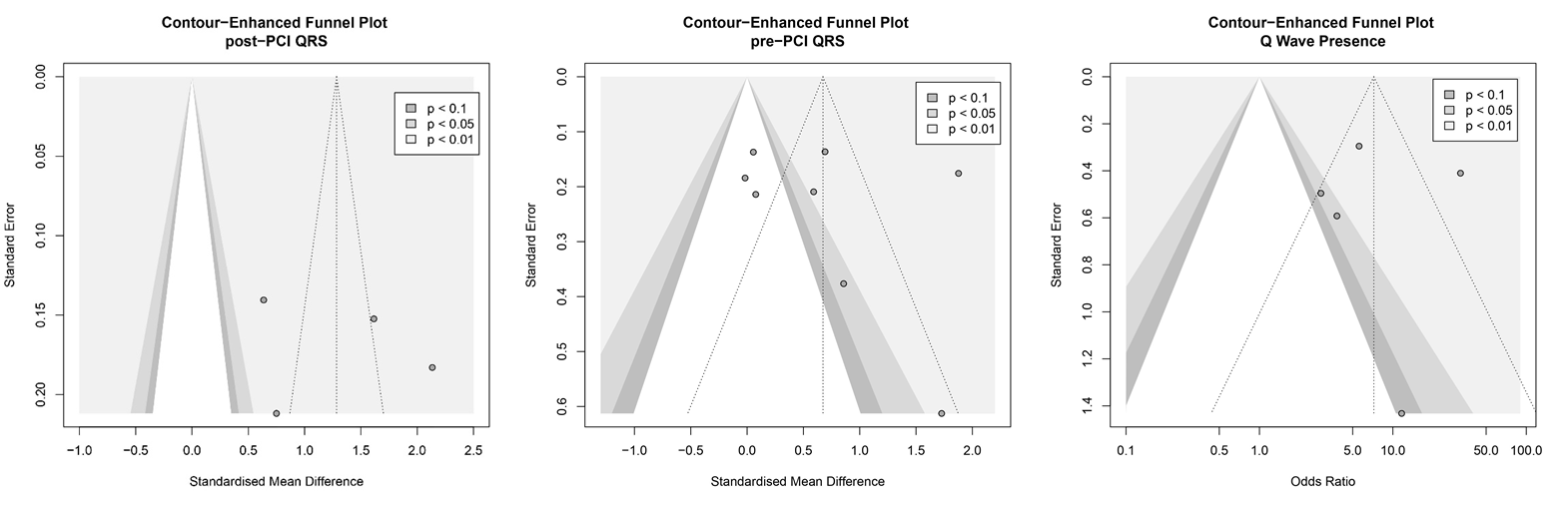
**

1. Page MJ, McKenzie JE, Bossuyt PM, Boutron I, Hoffmann TC, Mulrow CD, et al. The PRISMA 2020 statement: an updated guideline for reporting systematic reviews. International journal of surgery. 2021;88:105906.
